# Identifying and Removing Fraudulent Attempts to Enroll in a Human Health Improvement Intervention Trial in Rural Communities

**DOI:** 10.3390/mps7060093

**Published:** 2024-11-09

**Authors:** Karla L. Hanson, Grace A. Marshall, Meredith L. Graham, Deyaun L. Villarreal, Leah C. Volpe, Rebecca A. Seguin-Fowler

**Affiliations:** 1 Department of Public and Ecosystem Health, Cornell University, Ithaca, NY 14853, USA; gam263@cornell.edu (G.A.M.); lmc267@cornell.edu (L.C.V.); 2 Institute for Advancing Health Through Agriculture, Texas A&M AgriLife Research, Dallas, TX 75252, USA; meredith.graham@ag.tamu.edu (M.L.G.); deyaun.jafari@ag.tamu.edu (D.L.V.); r.seguin-fowler@ag.tamu.edu (R.A.S.-F.)

**Keywords:** data validation, fraud detection, research integrity, online survey research, research methods, online recruitment

## Abstract

Using the internet to recruit participants into research trials is effective but can attract high numbers of fraudulent attempts, particularly via social media. We drew upon the previous literature to rigorously identify and remove fraudulent attempts when recruiting rural residents into a community-based health improvement intervention trial. Our objectives herein were to describe our dynamic process for identifying fraudulent attempts, quantify the fraudulent attempts identified by each action, and make recommendations for minimizing fraudulent responses. The analysis was descriptive. Validation methods occurred in four phases: (1) recruitment and screening for eligibility and validation; (2) investigative periods requiring greater scrutiny; (3) baseline data cleaning; and (4) validation during the first annual follow-up survey. A total of 19,665 attempts to enroll were recorded, 74.4% of which were considered fraudulent. Automated checks for IP addresses outside study areas (22.1%) and reCAPTCHA screening (10.1%) efficiently identified many fraudulent attempts. Active investigative procedures identified the most fraudulent cases (33.7%) but required time-consuming interaction between researchers and individuals attempting to enroll. Some automated validation was overly zealous: 32.1% of all consented individuals who provided an invalid birthdate at follow-up were actively contacted by researchers and could verify or correct their birthdate. We anticipate fraudulent responses will grow increasingly nuanced and adaptive given recent advances in generative artificial intelligence. Researchers will need to balance automated and active validation techniques adapted to the topic of interest, population being recruited, and acceptable participant burden.

## 1. Introduction

Recruitment of participants into health improvement research studies has often utilized in-person approaches such as community events [1,2,3,4,5,6,7]. In 2020, the COVID-19 pandemic disrupted in-person recruitment and data collection for many researchers, and increased researchers’ reliance on online recruitment techniques and data collection approaches [8,9,10,11,12,13]. Using the internet for recruitment has been shown to be effective both in identifying underrepresented populations of interest and for broad recruitment of population-based samples [14,15,16,17,18]. However, as procedures become more automated, respondents can remain anonymous and may never interact with researchers or field staff [19].

Fraudulent responses to online surveys have been reported as a problem in a variety of disciplines. The volume of responses to online surveys that are suspected to be fraudulent is high: studies report 39% to 62% of survey attempts are fraudulent [19,20,21,22]. The highest levels of fraudulent response correspond to posting recruitment materials on social media [11,19,22,23], with one study highlighting that all cases identified via social media were fraudulent or suspicious [24]. Fraudulent responses can bias data, and automated and active techniques are used to try to screen out fraudulent responses. However, effective techniques may depend upon the topic of interest, population being recruited, participant burden, and compensation levels. Therefore, elaborating approaches to identify and remove fraudulent responses in a specific population may benefit future researchers.

This paper describes how we applied automated and active validation techniques to identify and remove fraudulent attempts to enroll in the Change Club (CC) study, a human health improvement trial launched in 13 medically underserved rural communities in New York and Texas [25]. The CC intervention was hypothesized to improve individual health and other outcomes among participants, their friends and family, and other community residents. Recruitment into the CC study occurred between June 2022 and May 2023 amid the COVID-19 pandemic state of emergency. Recruitment included centralized strategies such as online advertisements, mailing recruitment postcards and letters, and sending emails to purchased lists, as well as local strategies such as social media posts, traditional media (e.g., newspaper and TV), flyers and posters, extension educator actions (e.g., attending events, making phone calls), and referrals from friends and family members [26]. In the CC study, 23.5% of consented individuals (adults who gave informed consent to participate in the research study) reported that they heard about the study from friends or family and 20.5% via online information [26]. Given these predominant recruitment avenues, the potential for fraudulent attempts was particularly acute. Steps to identify and remove fraudulent attempts were developed based on the peer-reviewed literature regarding strategies for improving the trustworthiness of data collected via the internet [19,20,23,24,27]. As other authors have noted, each study needs to adapt existing recommendations to the target population and the unique needs of the study [19].

This paper has three objectives. First, we describe the dynamic four-stage process we developed for identifying potentially fraudulent responses during (1) recruitment and screening for eligibility and validation; (2) investigative periods requiring greater scrutiny; (3) baseline data cleaning; and (4) validation during the first annual follow-up survey. Second, we quantify the volume of fraudulent attempts identified by each action and the percentage removed from the sample. Third, we make recommendations for identifying fraudulent attempts in community-based health behavior research in rural areas and discuss some of the challenges to rigorous validation procedures.

## 2. Methods

### 2.1. Study Design

Recruitment into the CC study occurred from June 2022 through April 2023. Potential participants in the CC study first connected to an online automated eligibility screener in English on the Qualtrics survey platform (Qualtrics, Provo, UT, USA). To be eligible, individuals were required to be 18 or older, be a part of one of the 13 planned study communities, and (for CC members only) report one or more cardiovascular risk based on Life’s Simple 7 (e.g., high blood pressure, sub-optimal diet, low physical activity) [28,29]. The screener also collected contact information and demographic data on race, ethnicity, and sex.

Once identified as eligible, individuals were directed through the electronic study consent form which explained key aspects of the study and asked them to affirm that they understood and wished to participate. Once an individual consented, they were immediately directed to the baseline Qualtrics survey. To minimize item non-response, most questions in the survey required a response to advance in the survey. Several potentially sensitive questions (income, food security, relationship status, and social determinants of health) did not require a response. Questions relating to cancer, pregnancy, breastfeeding, and supplement use offered a response option of “prefer not to answer”. The survey was estimated to take 30 min to complete, and individuals were compensated USD 75 for annual survey completion [25]. CC members were also required to complete one 24 h dietary recall and to wear a pedometer for seven days and report daily steps.

### 2.2. Data Validation

Data validation occurred in four phases: (1) baseline validation procedures; (2) investigative procedures used during periods of high suspected fraud; (3) data cleaning procedures implemented at the conclusion of baseline data collection; and (4) an automated validation check (with active verification) employed during the first follow-up data collection period. Our consent documents communicated that “only valid responses will be accepted” and that “Your participation in this study may be stopped at any time by the investigator or sponsor without your consent. If the study team determines for any reason your safety or the scientific integrity of the study will be compromised by your continued participation in the study, you will be withdrawn”. Therefore, any cases identified as fraudulent were removed from the sample. All attempts barred from advancing in the study by any automated technique were provided with a message that included the central study email address which they could contact if they had questions or concerns.

Phase 1: Baseline Validation Protocol. This phase included automated and active techniques employed in real-time during recruitment and baseline data collection. Four automated techniques were employed to authenticate responses and automatically bar fraudulent attempts from enrolling. First, links to the eligibility screener posted on the study website were updated 3 to 5 times per week and access to the screener via outdated links was disabled, and links embedded in local recruitment materials were customized and monitored. Second, attempts were required to complete a Completely Automated Public Turing test to tell Computers and Humans Apart (reCAPTCHA), and scores < 0.5 were barred from continuing [30]. Third, attempts from IP addresses outside study locations and neighboring states were barred from continuing. This included any IP address not from NY, TX, neighboring states (LA, NM, OK, VT), or Ontario, Canada (adjacent to the upstate NY study location). Fourth, attempts that reported an email address identical to a previously consented individual were barred from continuing. Baseline validation also included an active check of remaining attempts to confirm that the mailing address was within a study state (NY or TX), and cases with addresses in other states were removed.

Phase 2: Investigative Procedures. During periods of high-volume suspected fraud, additional investigative techniques were used to identify fraudulent attempts. We defined “high-volume suspected fraud” as an observed surge in attempts of 20% or more higher than the average number of screeners completed in the past 3 days, or by observed social media post(s) by enrollees that encouraged others to provide false information in order to obtain compensation. During these periods, multiple attempts from a single IP address were barred from completing the screener. Other triggers for fraud investigation included a name, name embedded in an email address, or email “contact name” used for multiple attempts, or if key information (age, sex, race, or ethnicity) provided in the eligibility screener and survey was inconsistent.

Attempts during periods of high-volume fraud underwent two active investigative procedures. First, researchers validated addresses using Smarty^TM^ (Orem, UT, USA), a Coding Accuracy Support System (CASS)™ certified online tool to validate addresses against U.S. Postal Service data, which is updated monthly, and cases with invalid addresses were removed [31]. Second, researchers contacted remaining attempts via telephone to verbally verify or correct key information. Attempts were removed from the sample if (1) the phone number was invalid (e.g., wrong number, disconnected); (2) an individual could not be reached after three calls over a one-week period; (3) the individual did not respond within two weeks to a voicemail requesting that they call the office; and (4) the individual could not verify or correct their key information.

Phase 3: Data Cleaning Protocol. Data cleaning of baseline data from consented individuals included three steps: (1) double-checking key data; (2) double-checking for duplicate cases; and (3) applying ‘best practices’ for examining potentially fraudulent survey responses [19,27]. Because prior phases required researchers to vet thousands of attempts using active techniques in a fast-paced environment, data cleaning first involved double-checking four key items for all consented individuals: birthdate indicated age eligibility, survey was complete, street address was “valid” using Smarty^TM^, and zip code indicated a study location. Second, we double-checked for duplicate name, address, or phone number, and verified any suspect information with consented individuals. Cases that could not be verified using the procedures above were removed from the sample. Third, we applied a “3-strikes” rule [19,27] for identifying suspected fraudulent responses based on nine criteria: short response time (<15 min), low-probability responses (extreme height [32], body weight [33], BMI, or waist circumference [34], compared to national averages), internal inconsistencies between screener and survey (age, gender), low differentiation in response (90% or more of responses to 5-point scale items were equivalent), or repeat IP address. Consented individuals for whom three or more of these criteria applied were considered likely fraudulent and removed from the sample.

Phase 4: Follow-up Validation Protocol. During year-1 follow-up data collection, all consented individuals were asked to report their birthdate at the beginning of the Qualtrics survey (Qualtrics, Provo, UT), which was automatically validated against the birthdate reported at baseline. Cases with inconsistent birthdates were barred from continuing to data collection and were provided with the central study phone number to call if they believed an error was made. Consented individuals that could verify their name, phone number, street address, and birthdate were allowed to complete follow-up data collection. Some respondents described being reticent to record their actual birthdate for fear of identity theft. For example, one consented individual reported that they often used a standard “fake” birthdate, confirmed that specific date, provided their real birthdate, were allowed to continue to data collection, and the corrected birthdate was updated in the data. Also, if two of the three birthdate elements (month, day, year) matched and it seemed plausible that a typo had occurred, consented individuals were allowed to continue to data collection and the corrected birthdate was updated in the data. All consented individuals with inconsistent birthdates that could not be verified were removed from the sample.

Active data cleaning procedures were used to identify cases with either an implausible change in height (growth of 2+” or shrinkage of 4+”) or three or more low-probability responses to ten body measurements (extreme baseline height, baseline or time 1 body weight, or baseline or time 1 waist circumference; change in height more than ±1”; extreme change in body weight, BMI, or waist circumference; or BMI and waist circumference that changed in opposite directions). Extreme values for each variable were defined as both ±3 SD from the mean and also appeared as a break in the distribution on visual examination of boxplots. Researchers contacted identified cases via telephone to verbally verify name, mailing address, telephone, and height and weight at time 1, approximate change in height and weight over the past year, and approximate baseline values. Consented individuals were removed from the sample if (1) the phone number was invalid (e.g., wrong number, disconnected); (2) an individual could not be reached after three calls over a one-week period; (3) the individual did not respond within two weeks to a voicemail requesting that they call the office; or (4) the individual could not plausibly verify or correct (e.g., typo) body weight measurements.

### 2.3. Analysis

For each validation technique, the total number and percentage of attempts identified as invalid and excluded from the sample were calculated.

## 3. Results

Overall, 19,665 attempts to enroll in the CC study were recorded (Table 1). Across the four phases of automated and active validation procedures, over 14,633 attempts were identified as fraudulent (74.4%) and excluded from the study sample. 

Using techniques in the baseline validation protocol, 7792 attempts were identified as potentially fraudulent via automated techniques and were barred from enrollment (39.6%). Of these invalid attempts, most were attempts that originated from IP addresses outside study locations or neighboring states (4339; 22.1%) or could not pass reCAPTCHA screening (1985; 10.1%). A substantial number of attempts were also screened out because they used an expired link (977; 5.0%) or reported an email address identical to a prior consented individual (389, 2.0%). Using active techniques, another 102 attempts reporting a street address outside the study states were identified and barred from enrollment (0.5%).

Investigative procedures identified another 6626 attempts as fraudulent (33.7% of all attempts). During periods of high suspected fraud, multiple attempts from a single IP address were barred, which prohibited more than one thousand attempts from continuing (5.7%). Active investigation also identified cases with duplicate names or inconsistent information across the eligibility screener and survey, more than five thousand of which could not be validated with postal service records or verified with the individual (28.0%).

Data cleaning procedures identified 109 consented individuals as potentially fraudulent (0.6%) and they were removed from the sample. Only 15 of these removals were due to extreme or low-probability responses that triggered the “3-strikes” rule.

Validation of birthdate during follow-up data collection identified another 138 individuals as potentially fraudulent, all of whom were instructed to call the central study phone number if they believed an error was made. Fifty-eight of these individuals did call, and most (43, 74.1%) were able to provide verbal verification of birthdate and other key information. Ninety-five consented individuals could not verify key information or never called and were removed from the sample (0.5% of all attempts). Eleven consented individuals had an implausible change in height or at least three low-probability body measurement responses that could not be verified, and they were removed from the sample (0.1%).

## 4. Discussion

The CC intervention cRCT weathered a high volume of suspected fraudulent attempts—almost three-quarters of all attempts. Twenty-two percent of attempts were excluded as potentially fraudulent based solely on IP location which was fewer than reported in other studies [20]. This lower percentage may be due to the widespread availability of services to hide or change IP address, such as proxy servers or virtual private networks (VPNs) [35], which were not identified using our data validation protocols. Another 10% were excluded due to a low reCAPTCHA score, which was higher than reported in other studies [24,36,37]. These automated validation approaches are easy to implement, quick, and inexpensive. However, these steps did not screen out all fraudulent attempts. The active investigation technique of validating street addresses using Smarty^TM^, and verifying over the telephone any suspicious names, street addresses, and telephone numbers identified the most fraudulent attempts (28% of all attempts).

Recruitment into the CC study relied heavily on word-of-mouth and online recruitment techniques (23.5% and 21.6% of consented individuals, respectively) [26], both of which exposed this study to wide distribution as well as opportunism. For example, we observed a social media post by one individual encouraging others to lie in order to obtain compensation. In an effort to create friction for opportunists as well as bots or other automated attempts, we changed the study link frequently (daily during investigative periods) and altered social media posts to be images that included the study website URL (uniform resource locator) but did not provide a direct link. These actions may also create friction for real potential participants.

Compensation for this study (USD 75) was high relative to the effort required (an annual 30 min survey was the only activity for most consented individuals). It was also high relative to other studies that used similar methods of data collection [38]. Some researchers have suggested that remote work arrangements and economic downturn during the COVID-19 pandemic may have increased the prevalence of deceptive behavior to obtain research compensation [16]. Higher than average compensation may have exacerbated this behavior.

One aspect of the CC study was examination of how the strong social ties in rural areas may accelerate impacts to the community. To complete this analysis, we wanted to ensure that participants’ immediate family and other household members could enroll in the study. Therefore, we did not routinely bar multiple attempts from a single IP address. This choice was also noted in research involving couples who may share an IP address [19]. Furthermore, for many rural communities, the internet is unavailable, slow, or too expensive for many households [39,40]. In 2020, 22.3 percent of rural residents lacked home access to broadband internet [41]. Public internet access such as at libraries can fill this crucial gap. In this study, community members were offered the option to complete the eligibility screener, consent form, and survey at the local extension office, resulting in another instance in which multiple valid attempts might originate from a single IP address. However, during periods with a high volume of fraudulent attempts, we did bar multiple attempts from an IP address which excluded more than 1000 attempts from enrolling. As recruitment links were refreshed and the volume of fraudulent attempts waned, this restriction was removed. Community-based research or research in rural areas may need to balance the desire for broad inclusion of family members and lower-income residents who access the internet in public locations with the need to minimize fraudulent responses.

Active validation, investigation, and verification techniques were time-consuming. Researchers entered thousands of street addresses into the Smarty web interface multiple times per week during periods of active investigation. Other researchers have used similar techniques: Loebenberg and colleagues (2023) used address look-up [42] and Bowen and colleagues (2008) used reverse look-up of telephone numbers [43] as validation strategies. Active verification was even more time-consuming. To verify key information, including name, telephone number, and street addresses when fraud was suspected, we made more than 2000 phone calls. And for working phone numbers, researchers made up to three calls each before labeling the attempt as fraudulent. Phone verification of personal information has been performed in other studies where fraud was suspected [11,42]. If our study team reached an incorrect or disconnected phone number, we excluded the case, which is consistent with other studies that reported this as a common reason for exclusion [20].

Automated and active validation techniques were sometimes overly zealous. For example, five attempts that were initially eliminated during investigative procedures because the street address could not be validated subsequently called the researchers, verified a valid street address and other key information, and were allowed to advance to the consent phase. 58 people who did not pass automated validation of birthdate subsequently called the researchers and 43 of them could provide verification over the telephone (31.2% of all consented individuals who did not pass the automated birthdate validation protocol). Some individuals were also cautious that this might be a fraudulent study. A few respondents reported that they seldom use their real birthdate but rather a standard “fake” birthdate which they could verify against baseline data and was corrected. Thirty-two consented individuals were flagged as having an implausible change in height or at least three extreme or low-probability body measurements. Upon telephoning, researchers found that 20 of them (64.5%) could verify or plausibly correct typos in their body measurements and were retained in the study. When researchers have real interactions with individuals, it can buffer overly zealous automated validation, as well as screen out fraudulent attempts generated by automated techniques like bots.

## 5. Conclusions

This paper demonstrates the application of multiple approaches to fraud detection, data validation, and active data verification in a human health improvement intervention trial reliant on online and word-of-mouth recruitment, and it highlights some of the strengths and challenges of these approaches. Given recent advances in generative artificial intelligence (GenAI), fraudulent responses will grow increasingly nuanced and rapidly adaptive. Simple bots created using GenAI have already been shown to outperform common authentication methods like reCAPTCHA [44,45,46]. GenAI can also generate a unique open-ended response from the perspectives of individuals with specified demographics that are difficult to discern from those of real people [46]. In response to this and other related concerns, tools are emerging to aid researchers in GenAI fraud detection at scale (e.g., QualityScore) [47] and can be strengthened by concordant use of active verification such as the processes outlined in this paper. As these new tools and technologies evolve, we expect there will be a need for continued education by ourselves and the greater research community on best practices for handling potentially fraudulent data. Furthermore, researchers will need to adapt and balance automated and active validation techniques to consider the topic of interest, population being recruited, and feasible participant burden.

## Figures and Tables

**Table 1:** Methods to identify valid cases and number excluded by each validation technique.

Phase and Approach		Description of Techniques	Attempts Excluded	% Invalid	Total Count
**During recruitment and baseline data collection**	Eligibility screener attempted				19,665
	**Phase 1: Baseline Validation Protocol**				
	Automated validation	Attempted to use an expired link	−977	5.0	
		reCAPTCHA score < 0.5	−1985	10.1	
		IP addresses when completing eligibility screener **not** in NY, TX, or a neighboring state (LA, NM, OK, VT) or Ontario, Canada	−4339	22.1	
		Multiple attempts with same email address	−389	2.0	
		Eligibility screener abandoned (unable to determine validity)	−786	n/a	
	Active validation	Reported street address not in NY or TX	−102	0.5	11,087
	**Phase 2: Investigative procedures when fraud was suspected**				
	Automated investigation	▪ Multiple attempts from one IP address	−1129	5.7	
	Active investigation	▪ Reported street address was invalid ▪ Address or phone could not be verified	−5497	28.0	4461
	**Enrollment Procedures**				
	Automated enrollment procedures	▪ Screened as ineligible	−369	n/a	
		▪ Declined to consent	−1261	n/a	
		▪ Consented to participate		n/a	2831
**After baseline data collection**	**Phase 3: Data Cleaning Protocol**				
	Active data cleaning procedures	Re-checked key data for ineligible age or location, blank survey, or invalid address	−92	0.5	
		▪ Duplicate name, address, or phone # that could not be verified	−2	0.0	
		▪ Applied “3-strikes” rule using 9 criteria: 1. Short completion time 2–5. Low-probability response for 4 body measurement(s) 6–7. Inconsistencies (age, sex) 8. Low response differentiation in matrices 9. Duplicate IP address	−15	0.1	2722
**During Y1 data collection**	**Eligibility Verification**				
	Automated eligibility status check	▪ Study location inactive, cases dropped	−105	n/a	
		▪ Consented individual opted out of study	−33	n/a	
		▪ Consented individual moved and no longer eligible	−58	n/a	2526
	**Phase 4: Follow-up Validation Protocol**				
	Automated/active validation technique	▪ Follow-up DOB does not match baseline	−95	0.5	
		▪ DOB could not be verified			
	Active data cleaning procedures	▪ Implausible change in height ▪ Low-probability response for 3+ body measurements	−11	0.1	
		▪ Body measurements could not be verified			
**Cases retained for intervention trial**					2420

## Data Availability

The original contributions presented in the study are included in the article; further inquiries can be directed to the corresponding author.

## References

[1] Fam E., Ferrante J.M. (2018). Lessons learned recruiting minority participants for research in urban community health centers. J. Natl. Med. Assoc..

[2] Goldman V., Dushkin A., Wexler D.J., Chang Y., Porneala B., Bissett L., McCarthy J., Rodriguez A., Chase B., LaRocca R. (2019). Effective recruitment for practice-based research: Lessons from the REAL HEALTH-diabetes study. Contemp. Clin. Trials Commun..

[3] Guillory J., Wiant K.F., Farrelly M., Fiacco L., Alam I., Hoffman L., Crankshaw E., Delahanty J., Alexander T.N. (2018). Recruiting hard-to-reach populations for survey research: Using Facebook and Instagram advertisements and in-person intercept in LGBT bars and nightclubs to recruit LGBT young adults. J. Med. Internet Res..

[4] Safi A.G., Reyes C., Jesch E., Steinhardt J., Niederdeppe J., Skurka C., Kalaji M., Scolere L., Byrne S. (2019). Comparing in person and internet methods to recruit low-SES populations for tobacco control policy research. Soc. Sci. Med..

[5] Seguin R.A., Eldridge G., Graham M.L., Folta S.C., Nelson M.E., Strogatz D. (2015). Strong Hearts, healthy communities: A rural community-based cardiovascular disease prevention program. BMC Public Health.

[6] Seguin R.A., Morgan E.H., Hanson K.L., Ammerman A.S., Jilcott Pitts S.B., Kolodinsky J., Sitaker M., Becot F.A., Connor L.M., Garner J.A. (2017). Farm Fresh Foods for Healthy Kids (F3HK): An innovative community supported agriculture intervention to prevent childhood obesity in low-income families and strengthen local agricultural economies. BMC Public Health.

[7] Seguin R.A., Sriram U., Connor L.M., Silver A.E., Niu B., Bartholomew A.N. (2018). A civic engagement approach to encourage healthy eating and active living in rural towns: The HEART Club pilot project. Am. J. Health Promot..

[8] Hensen B., Mackworth-Young C., Simwinga M., Abdelmagid N., Banda J., Mavodza C., Doyle A., Bonell C., Weiss H. (2021). Remote data collection for public health research in a COVID-19 era: Ethical implications, challenges and opportunities. Health Policy Plan..

[9] Mitchell E.J., Ahmed K., Breeman S., Cotton S., Constable L., Ferry G., Goodman K., Hickey H., Meakin G., Mironov K. (2020). It is unprecedented: Trial management during the COVID-19 pandemic and beyond. Trials.

[10] Pocock T., Smith M., Wiles J. (2021). Recommendations for virtual qualitative health research during a pandemic. Qual. Health Res..

[11] Reed N.D., Bull S., Shrestha U., Sarche M., Kaufman C.E. (2024). Combating Fraudulent Participation in Urban American Indian and Alaska Native Virtual Health Research: Protocol for Increasing Data Integrity in Online Research (PRIOR). JMIR Res. Protoc..

[12] Seguin-Fowler R.A., Demment M., Folta S.C., Graham M., Hanson K., Maddock J.E., Patterson M.S. (2023). Recruiting experiences of NIH-funded principal investigators for community-based health behavior interventions during the COVID-19 pandemic. Contemp. Clin. Trials.

[13] Seguin-Fowler R.A., Eldridge G.D., Graham M., Folta S.C., Hanson K.L., Maddock J.E. (2023). COVID-19 Related Protocol Considerations and Modifications within a Rural, Community-Engaged Health Promotion Randomized Trial. Methods Protoc..

[14] Ali S.H., Foreman J., Capasso A., Jones A.M., Tozan Y., DiClemente R.J. (2020). Social media as a recruitment platform for a nationwide online survey of COVID-19 knowledge, beliefs, and practices in the United States: Methodology and feasibility analysis. BMC Med. Res. Methodol..

[15] Bragard E., Fisher C.B., Curtis B.L. (2020). “They know what they are getting into”: Researchers confront the benefits and challenges of online recruitment for HIV research. Ethics Behav..

[16] Bybee S., Cloyes K., Baucom B., Supiano K., Mooney K., Ellington L. (2022). Bots and nots: Safeguarding online survey research with underrepresented and diverse populations. Psychol. Sex..

[17] Musker M., Short C., Licinio J., Wong M.-L., Bidargaddi N. (2020). Using behaviour change theory to inform an innovative digital recruitment strategy in a mental health research setting. J. Psychiatr. Res..

[18] Watson N.L., Mull K.E., Heffner J.L., McClure J.B., Bricker J.B. (2018). Participant recruitment and retention in remote eHealth intervention trials: Methods and lessons learned from a large randomized controlled trial of two web-based smoking interventions. J. Med. Internet Res..

[19] Dewitt J., Capistrant B., Kohli N., Rosser B.S., Mitteldorf D., Merengwa E., West W. (2018). Addressing participant validity in a small internet health survey (The Restore Study): Protocol and recommendations for survey response validation. JMIR Res. Protoc..

[20] Ballard A.M., Cardwell T., Young A.M. (2019). Fraud detection protocol for web-based research among men who have sex with men: Development and descriptive evaluation. JMIR Public Health Surveill..

[21] Griffin M., Martino R.J., LoSchiavo C., Comer-Carruthers C., Krause K.D., Stults C.B., Halkitis P.N. (2022). Ensuring survey research data integrity in the era of internet bots. Qual. Quant..

[22] Pratt-Chapman M., Moses J., Arem H. (2021). Strategies for the identification and prevention of survey fraud: Data analysis of a web-based survey. JMIR Cancer.

[23] Vu M., Huynh V.N., Bednarczyk R.A., Escoffery C., Ta D., Nguyen T.T., Berg C.J. (2021). Experience and lessons learned from multi-modal internet-based recruitment of US Vietnamese into research. PLoS ONE.

[24] Pozzar R., Hammer M.J., Underhill-Blazey M., Wright A.A., Tulsky J.A., Hong F., Gundersen D.A., Berry D.L. (2020). Threats of bots and other bad actors to data quality following research participant recruitment through social media: Cross-sectional questionnaire. J. Med. Internet Res..

[25] Seguin-Fowler R.A., Hanson K.L., Villarreal D., Rethorst C.D., Ayine P., Folta S.C., Maddock J.E., Patterson M.S., Marshall G.A., Volpe L.C. (2022). Evaluation of a civic engagement approach to catalyze built environment change and promote healthy eating and physical activity among rural residents: A cluster (community) randomized controlled trial. BMC Public Health.

[26] Seguin-Fowler R.A., Graham M.L., Hanson K.L., Villarreal D.L., Eldridge G.D., Christou A., On A., Kershaw M., Folta S.C., Maddock J.E. Effective and Cost-Effective Strategies for Recruiting Rural Adults into a Civic Engagement and Health Behavior Change Research Study.

[27] Baker R., Downes-Le Guin T. Separating the wheat from the chaff: Ensuring data quality in internet samples. Proceedings of the The Challenges of a Changing World Proceedings of the Fifth ASC International Conference.

[28] Folsom A.R., Shah A.M., Lutsey P.L., Roetker N.S., Alonso A., Avery C.L., Miedema M.D., Konety S., Chang P.P., Solomon S.D. (2015). American Heart Association’s Life’s Simple 7: Avoiding heart failure and preserving cardiac structure and function. Am. J. Med..

[29] Ogunmoroti O., Allen N.B., Cushman M., Michos E.D., Rundek T., Rana J.S., Blankstein R., Blumenthal R.S., Blaha M.J., Veledar E. (2016). Association between Life’s Simple 7 and noncardiovascular disease: The Multi-Ethnic Study of Atherosclerosis. J. Am. Heart Assoc..

[30] Qualtrics Fraud Detection/Bot Detection. https://www.qualtrics.com/support/survey-platform/survey-module/survey-checker/fraud-detection/#BotDetection.

[31] Smarty: About Our Data. https://www.smarty.com/docs/our-data.

[32] *Table 205: Cumulative Percent Distribution of Population by Height and Sex: 2007 to 2008*; Statistical Abstract of the United States: 2011 (130th Edition); U.S. Census Bureau. https://www2.census.gov/library/publications/2010/compendia/statab/130ed/tables/11s0205.pdf.

[33] *Table 206: Cumulative Percent Distribution of Population by Weight and Sex: 2007 to 2008*; Statistical Abstract of the United States: 2011 (130th Edition); U.S. Census Bureau. https://www2.census.gov/library/publications/2010/compendia/statab/130ed/tables/11s0205.pdf.

[34] Ford E.S., Mokdad A.H., Giles W.H. (2003). Trends in waist circumference among US adults. Obes. Res..

[35] Wang J., Calderon G., Hager E.R., Edwards L.V., Berry A.A., Liu Y., Dinh J., Summers A.C., Connor K.A., Collins M.E. (2023). Identifying and preventing fraudulent responses in online public health surveys: Lessons learned during the COVID-19 pandemic. PLoS Glob. Public Health.

[36] Bonett S., Lin W., Sexton Topper P., Wolfe J., Golinkoff J., Deshpande A., Villarruel A., Bauermeister J. (2024). Assessing and Improving Data Integrity in Web-Based Surveys: Comparison of Fraud Detection Systems in a COVID-19 Study. JMIR Form. Res..

[37] Krawczyk M., Siek K.A. When Research Becomes All About the Bots: A Case Study on Fraud Prevention and Participant Validation in the Context of Abortion Storytelling. Proceedings of the Extended Abstracts of the CHI Conference on Human Factors in Computing Systems.

[38] Dominguez D., Jawara M., Martino N., Sinaii N., Grady C. (2012). Commonly performed procedures in clinical research: A benchmark for payment. Contemp. Clin. Trials.

[39] Graves J.M., Abshire D.A., Amiri S., Mackelprang J.L. (2021). Disparities in technology and broadband internet access across rurality: Implications for health and education. Fam. Community Health.

[40] Vogels E.A. Some Digital Divides Persist Between Rural, Urban and Suburban America. https://www.pewresearch.org/short-reads/2021/08/19/some-digital-divides-persist-between-rural-urban-and-suburban-america/.

[41] Federal Communications Commission (2020). 2020 Broadband Deployment Report.

[42] Loebenberg G., Oldham M., Brown J., Dinu L., Michie S., Field M., Greaves F., Garnett C. (2023). Bot or not? detecting and managing participant deception when conducting digital research remotely: Case study of a randomized controlled trial. J. Med. Internet Res..

[43] Bowen A.M., Daniel C.M., Williams M.L., Baird G.L. (2008). Identifying multiple submissions in Internet research: Preserving data integrity. AIDS Behav..

[44] Cleary M., Kornhaber R., Le Lagadec D., Stanton R., Hungerford C. (2024). Artificial intelligence in mental health research: Prospects and pitfalls. Issues Ment. Health Nurs..

[45] Godinho A., Schell C., Cunningham J.A. (2020). Out damn bot, out: Recruiting real people into substance use studies on the internet. Subst. Abus..

[46] Irish K., Saba J. (2023). Bots are the new fraud: A post-hoc exploration of statistical methods to identify bot-generated responses in a corrupt data set. Personal. Individ. Differ..

[47] Crothers E.N., Japkowicz N., Viktor H.L. (2023). Machine-generated text: A comprehensive survey of threat models and detection methods. IEEE Access.

